# Long noncoding RNA MALAT1 promotes cardiomyocyte apoptosis after myocardial infarction via targeting miR-144-3p

**DOI:** 10.1042/BSR20191103

**Published:** 2019-08-02

**Authors:** Xiaohong Gong, Yun Zhu, Haixia Chang, Yongqin Li, Feng Ma

**Affiliations:** 1 Department of Cardiovascular Medicine, Xi’an Central Hospital, Xi’an City 710003, Shaanxi Province, P.R. China; 2 Department of ENT, the First Affiliated Hospital of Xi’an Jiao Tong University, Xi’an City 710061, Shaanxi Province, P.R. China; 3 Department of Cardiovascular Medicine, the second Affiliated Hospital of Xi’an Jiao Tong University, Xi’an City 710003, Shaanxi Province, P.R. China

**Keywords:** cardiomyocyte apoptosis, long non-coding RNA MALAT1, miR-144-3p, Myocardial infarction

## Abstract

Our study aims to excavate the role of metastasis-associated lung adenocarcinoma transcript 1 (MALAT1) in myocardial infarction (MI), especially in an ischemia/reperfusion injury model and the underlying mechanism involving the MALAT1-miR144 axis. Our results demonstrated that the expression of MALAT1 has a higher level, while miR-144 expression significantly reduced in myocardial tissue after MI and also in left anterior descending (LAD)-ligation mice. This result was confirmed *in vitro* studies in HL-1 cardiomyocytes followed with hypoxia/reoxygenation. In addition, overexpression of MALAT1 by MALAT1-pcDNA injection into the mice with LAD increased myocardial apoptosis *in vivo*, while this effect was attenuated by miR-144 mimic. Bioinformatics analysis exhibits that 3′-UTR of MALAT1 is targeted to the miR-144-3p. Up-regulation miR-144 blunted the hypoxia- or MALAT1-induced cell apoptosis. In conclusion, the expression of MALAT1 was increased, whereas miR-144 expression was down-regulated in the myocardium after AMI. MALAT1 up-regulation plays a critical role in promoting cardiomyocytes apoptosis via targeting miR-144.

## Introduction

Myocardial infarction (MI), is a cardiovascular disorder that occurs when the blood flow is decreased or stopped to part of the heart, which renders the heart muscle damage [1]. The incidences of the MI are still increasing worldwide each year. MI is widely considered to be happened due to coronary artery diseases which are characterized with the inflammatory response and apoptosis of cardiomyocytes caused by exposure to prolonged ischemia after coronary artery occlusion [2–4]. It has been reported that reperfusion after ischemia induces additional cell apoptosis and also increases the infarct size, which is named as myocardial ischemia/reperfusion (I/R) injury [5]. The risk factor for MI includes high blood pressure, obesity, smoking and so on [6,7]. The underlying mechanism for MI is the complete blockage of coronary artery by the rupture of the atherosclerotic plaque [4]. Since MI affects human health by inducing a very high morbidity and mortality rate all over the world, exploring the new diagnosis and treatment target is urgently needed.

Recently, noncoding RNAs have been reported involved in the MI pathogenesis and progression [8]. Among these noncoding RNAs, long noncoding RNAs (lncRNAs), which are a group of noncoding RNAs with more than 200 nucleotides in length, were reported to play important roles in the development of different type of human diseases, including cancer. MI, et al reviewed the evidence linking lnRNAs to diverse human diseases, and indicated that LncRNAs played important roles in human diseases, through regulation of physiological processes, including cell proliferation, differentiation, inflammation and cell apoptosis [9]. Several studies proved that the aberrant expression of lncRNAs can be used as a biomarker and plays critical roles in cardiac development and cardiovascular disease. LncRNA AK088388 was reported to promote autophagy via direct binding of miR-30a to affect myocardial I/R injury [10]. Xu et al. reported that lncRNA SLC8A1-AS1 could activate the cGMP-PKG signaling pathways to protect myocardial damage in mouse MI model [11]. Moreover, down-regulation of lncMIRT1 improved the I/R-induced injury in aged diabetic rat model though inhibition of NFκB signaling pathway [12]. All these studies indicated lncRNAs affects the pathogenesis, development and progression of MI and suggested their potential to be explored as diagnosis biomarker or therapeutic target for MI treatment.

LncRNA, MALAT1 (metastasis-associated lung adenocarcinoma transcript 1), located at 11q13, with a length of 8000 base pair, was firstly reported to participate in the progression of non-small-cell lung cancer (NSCLC) and affect NSCLC cell metastasis [13]. Later, lncRNA MALAT1 was reported to regulate the vessel growth and endothelial cell function [14]. Recently, several researchers found that aberrant expression of MALAT1 plays roles in cardiovascular disease. Hua Li et al. reported that rs619586AG/GG genotypes in *MALAT1* may protect against the occurrence of CAD [15]. Another study demonstrated that MALAT1 was up-regulated in ischemia model and played anti-apoptotic and anti-inflammatory role in *in vivo* and *in vitro* model [16]. However, the detailed mechanism of MALAT1 regulation on the pathogenesis of MI remains largely unknown. Our study aims to explore the role of MALAT1 in MI, especially in an I/R injury model and the underlying mechanism involving the MALAT1-miR144 axis. Our findings revealed that MALAT1 was up-regulated, while the expression level of miR-144 has a significant decline in myocardial tissue after AMI. *In vitro* studies, we confirmed this result in HL-1 cardiomyocytes followed with hypoxia/reoxygenation (H/R). We further proved that MALAT1 promoted myocardial apoptosis via direct binding of miR-144-3p. In conclusion༌MALAT1-miR144 axis has the potential biomarker for diagnosis of MI, as well as the potential therapeutic target for treatment of MI.

## Materials and methods

### Patients

A total of 40 patients who underwent coronary angiography to assess chest pain were enrolled in the First Affiliated Hospital of Xi’an Jiao Tong University, China. The MI was diagnosed using electrocardiographic criteria while non-STEMI was diagnosed based on the elevated serum troponin levels in addition to clinical symptoms consistent with cardiac ischemia. Angina was characterized by no elevated serum troponin levels and no ST segment elevation on electrocardiograph. Patients with a history of hepatitis, hepatic failure, end-stage renal failure, cardiomyopathy, congenital heart disease, hemorrhage, previous thoracic irradiation therapy or malignant disease cannot participate in the present study. Twenty healthy persons were enrolled as normal control. All the protocols used in study were approved by the First Affiliated Hospital of Xi’an Jiao Tong University, and informed consent was signed by each participant.

### *In vivo* I/R

All procedures used in the present study were approved by the First Affiliated Hospital of Xi’an Jiao Tong University, 4-6 months old male mice, were anesthetized using ketamine and xylazine (120 mg/kg body weight and 5 mg/kg body weight, respectively) by intraperitoneal injection and then subjected to left anterior descending (LAD) coronary artery occlusion followed by reperfusion (LAD/reperfusion) as previous studies [17]. Briefly, locate the LAD on the surface of the heart by using a dissection microscope and the LAD was ligated for 30 min. After ligation, the blood flow was restored. Sham animals were subjected to the surgery without LAD occlusion. Forty-eight hours later, the mice were anesthetized with isoflurane (4% to induce and 1.5–2% to maintain anesthesia, inhaled via a nose cone) during the whole procedure and 2-dimensional echocardiography was performed on a Vevo 2100 System (Visual Sonics, Toronto, Ontario, Canada). Mice were randomly divided into four groups: sham, cardial I/R model, sham+shMALAT1 and I/R + shMALAT1. With the help of 27 gauge needel, 200 nM of shMALAT1 in the tuberculin syringe was inserted into the second left intercostals space of animal. When the pulsatile blood appeared in the needle hub, sh-MALAT1 was injected in a continuous and slow motion.

### Cell cultures

Mouse cardiomyocyte cell line HL-1(Novobio Inc., Shanghai, China) was maintained in the Claycomb medium (Sigma–Aldrich, U.S.A.) supplemented with 10% fetal bovine serum, 100 units/ml penicillin, 100 mg/ml streptomycin, 0.1 mM norepinephrine and 2 mM L-glutamine. Experimental cell model was developed by exposing of the cell to 10 h of hypoxia condition with 95% N_2_, 5% CO_2_, and then reoxygenation was performed by exposure with 2 h of 95% O_2_, 5% CO_2_.

### Quantitative real-time PCR

Total RNA was extracted using the TRIzol1Plus RNA Purification Kit (TARARA, China). Reverse transcription was performed by using 2 µg of RNA with reverse transcriptase. Then qRT-PCR was conducted to detect the relative expression of MALAT1 and miR-144 with 7500 Real-Time PCR System (Life Technologies, U.S.A.) by using the SYBR Premix Ex Taq II (TAKARA, China) with their primers. GAPDH or U6 were used as the internal reference for MALAT1and miR144. MALAT1 primers were forward: 5′-AAAGCAAGGTCTCCCCACAAG-3′, reverse: 5′-GGTCTGTGCTAGATCAAAAGGCA-3′; miR-144 forward: 5′-CCTCGCACCTGGAGGCTGGCTG -3′; reverse: 5′-TTATCAGTTGGGAAAATAGTTA -3′

### Transfection

HL-1 cells were seeded into 6-well plates and cultured for overnight. Then cells were transfected with siRNAs or overexpression plasmids using Lipofectamine RNAiMAX reagent (Life Technologies, U.S.A.). ShMALAT1 or small interfering RNA against miR-144 (miR-144 inhibitor), or the scrambled negative control (negative control) were used to knockdown of MALAT1 or miR-144, respectively by transfection. Overexpression of MALAT1 was achieved by transfection of episomal pcDNA plasmids (only pcDNA vector or pcDNA containing MALAT1) using Lipofectamine 2000 reagent (LifeTechnologies). Forty-eight hours after transfection, cells were collected and total RNAs were extracted for RT-qPCR analysis; the other part of the harvested cells were used for flow cytometry analysis. The miR-144 inhibitor and miR-144 mimic oligonucleotides were purchased from GenePharma (Shanghai, China).; For MALAT1 overexpression, the full-length human MALAT1 cDNA was amplified using the following primers: forward, 5′-GGCGGTACC ATGAAACAATTTGGAGAAG -3′; reverse, 5′-GCGCTCGAG CTAAGTTTGTA-CATTTTGCC -3′. The cDNA product was subcloned into the pcDNA3.1(+) mammalian expression vector (Invitrogen) at the *KpnI* and *XhoI* sites to generate pcDNA3.1-MALAT1 and stable colonies were screened using G418. The pcDNA3.1(+) vector was used as negative control (pcDNA3.1-NC). For MALAT1 knockdown, two MALAT1-targeting shRNAs (MALAT1-shRNAl, 5′- TCCACTT-GATCCCAACTCATC-3′; MALAT1-shRNA2, 5′- TTCCTTAGTTGGCATCAAGGC-3′) and a scrambled shRNA (scr, 5′-GACCTGTACGC-CAACACAGTG-3′) used as negative control were synthesized at Genechem (Shanghai, China). The shRNAs were subcloned into the psilencer 4.1 CMV neo vector (Invitrogen) followed by selection with puromy-cin (Gibco, Grand Island, NY, U.S.A.) to generate stable cell lines. All plasmids were isolated using the DNA Midiprep kit (Qia-gen, Germany).

### Western blot

Cells were harvested and cell lysates were collected by digestion of cells with RIPA Buffer (Beyotime, China). The concentration of total proteins for each sample was quantified using BCA kit (Solarbio, Beijing, China) and 30 μg of each sample was then separated by SDS–PAGE gel. Then the proteins were transferred onto PVDF membranes. The membranes were blocked with 5% skim milk at room temperature for 1 h and then the membranes were incubated with primary antibodies, including anti-cleaved-caspase3 (CST, U.S.A.), cleaved-PARP (Abcam, Cambridge, U.K.) and anti-GAPDH (Beyotime, China) antibodies for overnight at 4°C. After washing with TBS containing 0.1% Tween-20 for four times and 5 min for each time, the membrane was incubated with anti-rabbit immunoglobulin G (IgG)-HRP conjugate. Protein expressions were finally visualized using enhanced chemiluminescence system (Beyotime, China).

### Flow cytometry to detect cell apoptosis

The digested cells were collected and resuspended in 100 μl of binding buffer containing of 5 μl Annexin V-FITC and 5 μl propidium iodide (PI). Then stained at room temperature avoiding of light and incubated for 20 min. Then, the flow cytometry analysis was performed to determine the cell apoptosis with flow cytometer (Beckman Coulter, Miami, FL, U.S.A.).

### Statistical analysis

The data were analyzed with Graphpad prism 7 software and the data were presented as the mean ± standard deviation (SD). The student’s *t* test was used for the comparisons between two groups and the one-factor analysis of variance (ANOVA) method was used for the comparisons among multiple groups. A probability value of *P*<0.05 was considered as the statistically significant difference.

## Results

### The expression of MALAT1 was increased while the expression of miR-144-3p mRNA levels was decreased in AMI patients and mice LAD models

**Figure 1:**
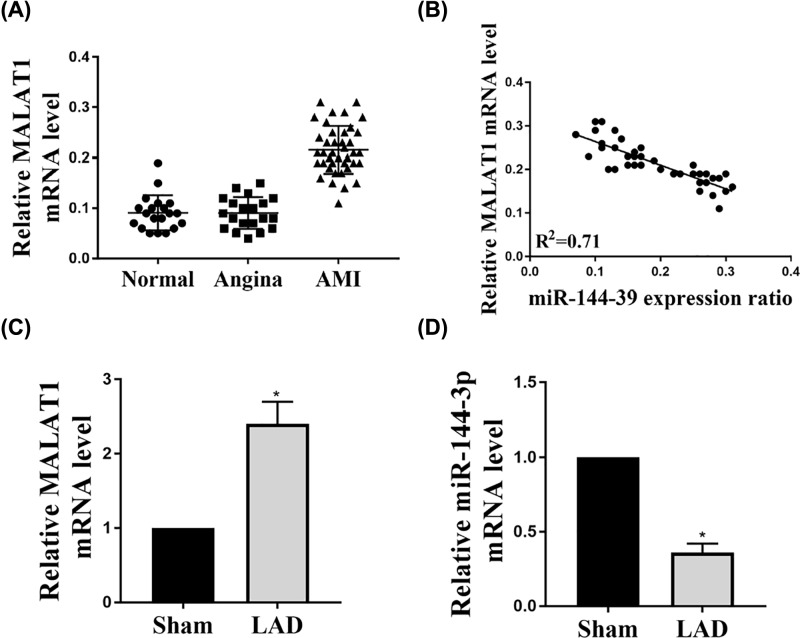
The expression of MALAT1 and miR-144-3p mRNA levels in AMI patients and mice LAD models (**A**) Total RNAs were extracted from the peripheral venous blood from each subject in Normal, Angina and AMI group. The relative expression levels of MALAT1 in AMI patients (*n*=40), normal control (*n*=20) and angina patient (*n*=20) were determined by qRT-PCR. (**B**) The relationship of MALAT1 and miR-144-3p expression was analyzed. (**C,D**) Male mice were subjected to LAD coronary artery occlusion followed by reperfusion (LAD/reperfusion). The expressions of MALAT1 and miR-144 mRNA in heart tissue were determined by real-time PCR. The results were presented from three independent experiments with the similar results. *P<0.05.

Total RNAs were extracted from peripheral venous blood from each subject in Normal, Angina and AMI group. The relative expression levels of MALAT1 in AMI patients (*n*=40), normal control (*n*=20) and angina patient (*n*=20) were determined by qRT-PCR. The results indicated that MALAT1 expression in AMI (*n*=40) was increased compared with angina (*n*=20) and normal control (*n*=20) (Figure 1A). And the expression of MALAT1 has a negative correlation with the expression of miR-144-3p levels (Figure 1B). *In vivo* LAD-induced I/R injury model was established in mouse and then the expression of MALAT1 and miR-144 in the cardiac infarction sites were measured by qRT-PCR. These findings indicated that the expression of MALAT1 has a higher level, while miR-144 expression significantly reduced in the mice heart of AMI (Figure 1C,D). Taken together, MALAT1 expression was up-regulated, while the expression of miR-144-3p mRNA levels was decreased and their expression was negatively correlated in AMI patients and mice LAD models.

### MALAT1 regulates cardiomyocytes apoptosis in HL-1 cells

**Figure 2:**
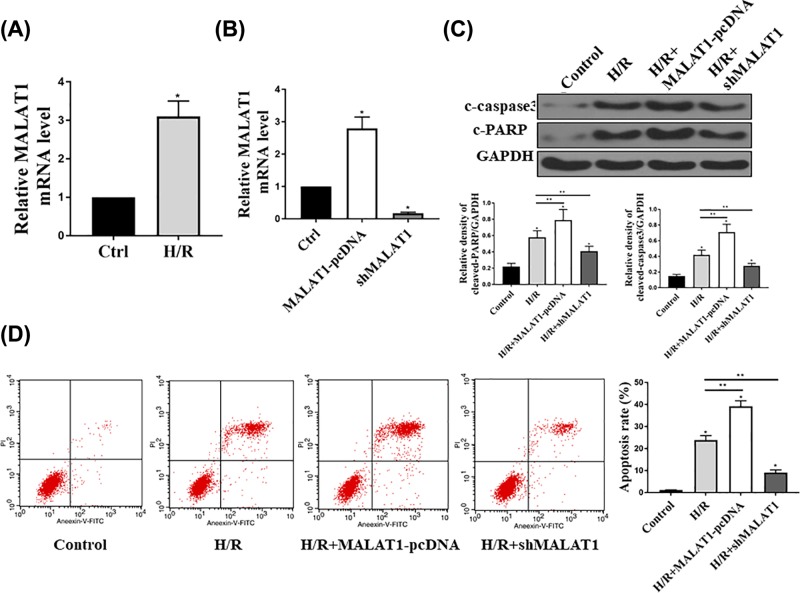
MALAT1 regulates cardiomyocytes apoptosis in HL-1 cells Cells were subjected to 10 h of hypoxia with 95% N_2_, 5% CO_2_, and subsequent 2 h of reoxygenation with 95% O_2_, 5% CO_2_; (**A**) Total RNAs were extracted from control (Ctrl) and H/R group were reverse transcribed into cDNA. Then qRT-PCR was performed to examine the relative expression of MALAT1. (**B**) HL-1 cells were transfected with pcDNA-MALAT1 or shMALAT1, and then the expression levels of MALAT1 were examined by qRT-PCR. (**C**) Western blot assay was performed to examine the cleaved caspase 3 and cleaved PARP in in control, H/R cell, H/R cell transfected with MALAT1-pcDNA and shMALAT1 and the expression levels of c-caspase3 and c-PARP were quantified. (**D**) Annexin V and PI double staining was conducted in control, H/R cell, H/R cell transfected with MALAT1-pcDNA and shMALAT1 and then flow cytometry assay was performed to determine the cell apoptosis. The experiments were repeated for at least three times. *n*=5, *P<0.05; **P<0.01.

We examined the mRNA expression levels of MALAT1 in HL-1 cardiomyocytes followed with H/R. As shown in Figure 2A, consistent with the *in vivo* results, H/R induced up-regulation of MALAT1 expression. Next, MALAT1 overexpression or knockdown were achieved by transfection of cells with MALAT1-pcDNA or shMALAT1 and then the MALAT1 expression levels were detected by qRT-PCR as shown in Figure 2B. Then we examined the effects of the expression of MALAT1 on cell apoptosis. H/R treatment induced the apoptosis by elevating the expression of cleaved caspase3 and cleaved PARP. Up-regulation of MALAT1 enhanced the H/R-induced cell apoptosis, yet, knockdown MALAT1 might attenuate the H/R-induced cell apoptosis by inhibition of the expression of cleaved caspase3 and PARP (Figure 2C). Moreover, flowcytometry results further confirmed the promotion role of MALAT1 on cell apoptosis (Figure 2D). Therefore, all results proved MALAT1 acts a promotion factor in the regulation of cardiomyocytes apoptosis in HL-1 cells.

### Down-regulation of MALAT1 reduced myocardium apoptosis in LAD mice

**Figure 3:**
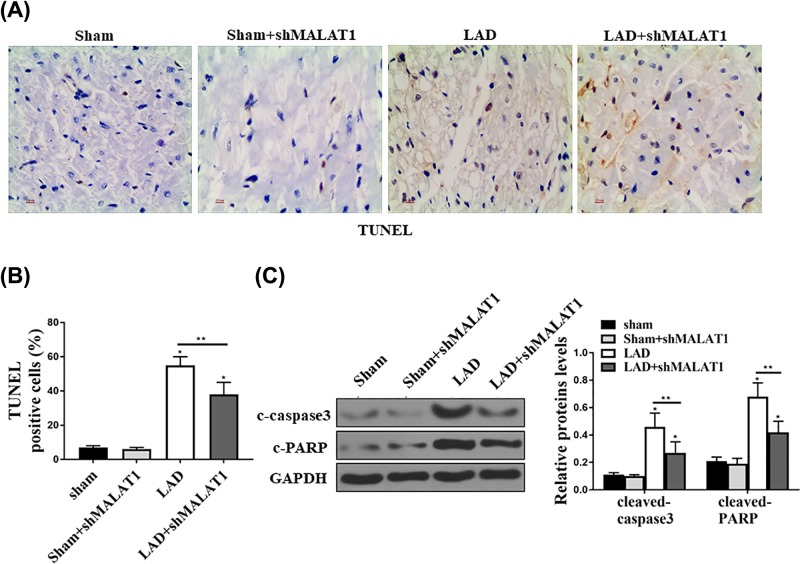
Inhibition of MALAT1 reduced myocardium apoptosis in LAD mice Mice injected with shMALAT1 followed with I/R injury. (**A**) TUNEL staining assay was performed to detect the apoptosis in cardiac infarction sites. (**B**) The TUNEL positive cells were counted and analyzed. (**C**) Western blot assay was used to detect the c-casepase3 and c-PARP in the cardiac infarction sites and the expression levels were quantified. All the experiments were repeated for at least three times and got the similar results. *n*=5, *P<0.05; **P<0.01.

We next examined whether intravenous administration of adMALAT1 could reduce apoptosis after AMI in mice. After mice were subjected with LAD-induced I/RI/RI/R injury, we injected animals with adMALAT1. Forty-eight hours later, the mice hearts were isolated for TUNEL staining and protein expression analysis. The TUNEL-positive cells in LAD groups were higher, and down-regulation of MALAT1 with shMALAT1 reduced LAD-induced apoptosis (Figure 3A,B). Then, we examined the expression of cleaved caspase3 and PARP to determine the apoptosis by Western blot. We found LAD significantly reduced the apoptosis by reducing the protein expression of cleaved caspase3 and PARP, whereas down-regulation of MALAT1 with shMALAT1 significantly attenuated LAD-induced cell apoptosis (Figure 3C). Altogether, these data demonstrated that down-regulation of MALAT1 reduced the myocardium apoptosis in LAD mice.

### MALAT1 targets and regulates miR-144-3p expression

**Figure 4:**
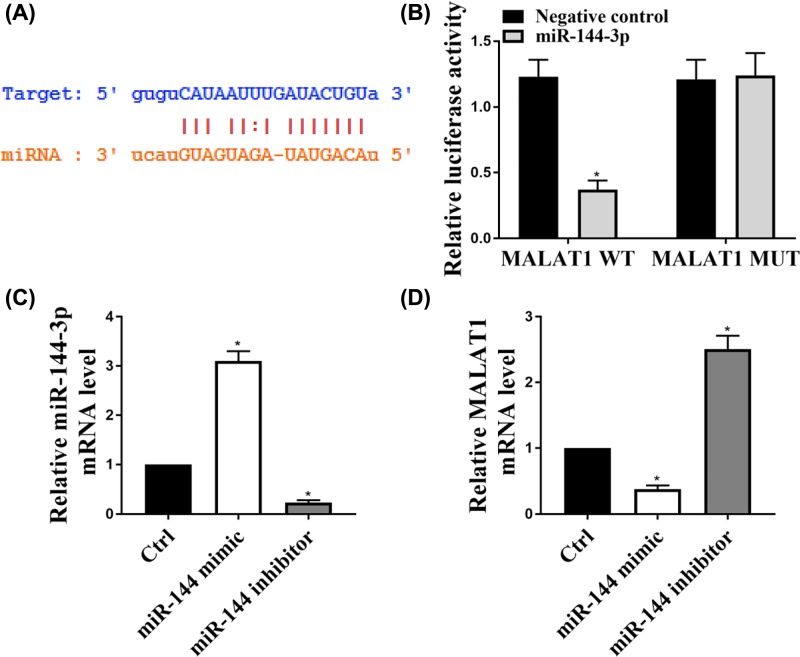
MALAT1 targets and regulates miR-144-3p expression (**A**) The putative target sequence for miR-144-3p on the 3′-UTR of MALAT1 was predicted by TargetScan prediction software. (**B**) The relative luciferase activity of WT or MUT MALAT1 3′-UTR reporter plasmids was detected by luciferase reporter assay after the cells transfected with miR-144-3p. (**C**) HL-1 cells were transfected with miR-144-3p mimic or miR-144-3p inhibitor and then the expression of miR-144 was detected by qRT-PCR. (**D**) The expression of MALAT1 was detected by qRT-PCR in the cells transfected with miR-144-3p mimic or miR-144-3p inhibitor. All the results were presented the data from three independent experiments with the similar results. *n*=5, *P<0.05.

The putative target sequence for miR-144-3p on the 3′-UTR of MALAT1 was predicted by TargetScan software. Figure 4A showed the predicted binding sites. Then dual luciferase assay was conducted by the miR-144 mimic co-transfected with pc-MALAT1 wild-type (WT) or pc-MALAT1 mutant (MUT) in HL-1 cells. We found that the miR-144 reduced the luciferase activity in MALAT1 WT transfected cells, but not in MALAT1 MUT cells (Figure 4B). We next explored whether the MALAT1 expression was influenced by the levels of miR-144 in HL-1 cells. The expression of miR-144 mRNA levels was shown in Figure 4C. As shown in Figure 4D, overexpression of miR-144 decreased MALAT1 expression, while, down-regulation of miR-144 induced MALAT1 expression. These results indicated miR-144-3p,as a direct target of MALAT1, regulated MALAT1 expression.

### The effect of MALAT1 on cardiomyocytes apoptosis was mediated by miR-144

**Figure 5:**
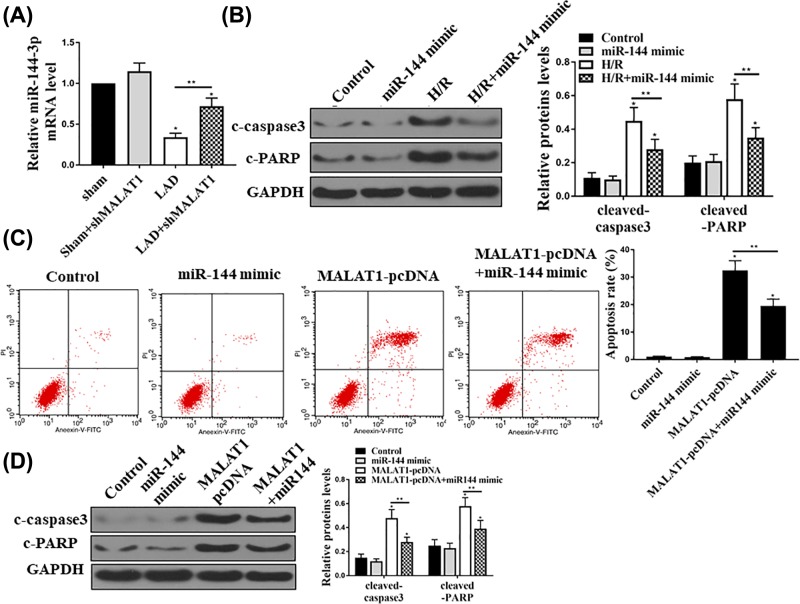
The effect of MALAT1 on cardiomyocytes apoptosis was mediated by miR-144 (**A**) Mice injected with shMALAT1 followed with I/R injury. MiR-144 mRNA levels in the indicated each group was examined by qRT-PCR. (**B**) The cleaved caspase3 and PARP in each group were examined by Western blot assay in HL-1 cells. (**C**) Annexin V and PI double staining were performed then the cell apoptosis rate in each group were determined and analyzed by flow cytometry in HL-1 cells. (**D**) HL-1 cells were transfected miR-144-3p mimic, MALATA-pcDNA or miR-1444 together with MALAT1, and then Western blot was performed to check the protein expression of cleaved caspase3 and PARP. All the results presented the data from three independent experiments with the similar results. *n*=5, *P<0.05; **P<0.01.

To determine whether MALAT1-induced apoptosis was mediated by miR-144, we first detected the miR-144 mRNA levels in the mice with LAD. LAD reduced miR-144 expression, while shMALAT1 pretreatment increased the miR-144 expression (Figure 5A). *In vitro*, we first found overexpression of miR-144 inhibited H/R-induced apoptosis by reducing the expression of cleaved caspase3 and PARP, determined by Western blot assay (Figure 5B). In addition, we further found that overexpression of miR-144 also reduced MALAT1-induced apoptosis. As shown in Figure 5C, apoptosis cell rate was increased after MALAT1 overexpression, while the increased apoptosis cell rates were reduced by miR-144 mimic treatment. Moreover, Western blot results showed that the cleaved caspase3 and PARP protein levels increased by the MALAT1, while overexpression of miR-144 reduced their expressions (Figure 5D). Thus, these results suggested that the effect of MALAT1 on cardiomyocytes apoptosis was mediated by miR-144.

## Discussion

More and more studies showed that lncRNAs play critical roles in physiological and pathological responses in different human disease. However, their role in cardiovascular disease is not fully investigated. Recently, lncRNA FosDT was reported to be induced in adult rat with transient cerebral artery occlusion and knockdown of FosDT plays a protect role against infarct tissue injury [18]. Another lncRNA named Chear was found to be highly induced in the heart of mice with pressure-overload-induced heart failure and specifically expressed in cardiomyocytes. Chear knockout significantly attenuated the transverse aortic constriction-induced cardiac fibrosis and hypertrophy in the mouse model [19]. LncRNA MALAT1, which was firstly reported to participate in the progression of NSCLC and affect NSCLC cell metastasis [13], was recently revealed plays important role in in cardiovascular disease. It was proved to be up-regulated in MI and played a protection role through regulation of the apoptosis in the *in vivo* and *in vitro* MI models [15]. However, the detailed biological function and the involved mechanism of its regulation of pathogenesis and development of MI are still under investigation.

Our study tried to comprehensively explore the role of lncRNA MALAT1 and its biological effect in MI *in vivo* and *in vitro*. We first assess the expression-levels of MALAT1 in MI patient samples and these results indicated that expression of MALAT1 was significantly up-regulated in both MI patients and mice LAD models compared with normal control. Besides MALAT1, we also detect the expression of small noncoding microRNA miR-144-3p. It has been reported to participate in the development of different type of cancers. However, its function in cancer is still controversial. Some studies showed that miR-144-3p could not only inhibite the tumor cell proliferation but also induce apoptosis, indicating its role as a tumor suppressor in multiple myeloma and laryngeal squamous cell carcinoma [20,21]. Yet, in thyroid carcinoma, miR-144-3p promotes the tumor progression [22]. Recently, miR-144-3p was proved to play a protection role in I/R by reducing I/R-induced cardiomyocytes death via targeting CUGBP-COX2 pathway, indicating its involvement in MI [23]. In our study, qRT-PCR results revealed that the expression of miR-144-3p was markedly reduced in MI patients and mice LAD models, compared with normal control. The elevated MALAT1 expression and knockdown miR-144-3p expression was further confirmed in the H/R-treated HL-1 cell *in vitro*. These results suggested that not only MALAT1 but also miR-144-3p have the potential to be served as biomarker for the diagnosis of MI.

Since cardiomyocytes apoptosis is a characteristic of MI and I/R(I/R), inhibition of the cardiomyocytes apoptosis will be a big interest for treatment of MI [24]. According to our results, they indicated that up-regulation of MALAT1 promoted the cardiomyocytes apoptosis, whereas down-regulation of MALAT1 reduced the cardiomyocytes apoptosis by decreasing the expression of apoptotic markers that include cleaved caspase3 and cleaved PARP. This novel finding suggested the MALAT1 could be a therapeutic target for the treatment of MI by inhibition of the expression of MALAT1. Moreover, informatic predication software predicts the binding site at 3′TUR of MALAT1 with miR-144-3p. Dual luciferase assay further confirmed their direct binding. In addition, qRT-PCR data demonstrated that MALAT1 expression was negatively regulated by miR-144-3p. Since it was previously proved to play a protection role in MI by inhibiting the cardiomyocytes apoptosis [23], we next investigate whether MALAT1-induced cardiomyocytes apoptosis was regulated by miR-144-3p. Both Western blot and flow cytometry analysis elucidated the cardiomyocytes apoptosis was induced by the MALAT1, while overexpression of miR-144 reduced the cardiomyocytes apoptosis. Thus, these results suggested that the effect of MALAT1 on cardiomyocytes apoptosis was mediated by miR-144. This is the first reported to excavate the MALAT1/miR-144-3p axis in MI and the novel findings uncovered the basis of how this axis regulated the cardiomyocytes apoptosis and proved its potential to be explored not only for the biomarker for the diagnosis but also the potential to be the therapeutic target to treatment of MI. However, the patient sample scale is still limited and majority of the mechanistic studies were established in either mouse model or H/R-induced cells. In the future, large scale patient samples are needed to confirm the biomarker role of this MALAT1/miR-144-3p axis and also more mouse models are needed to be employed to verify its potential as therapeutic target to treat MI.

## Abbreviations

H/R: hypoxia/reoxygenation
I/R: ischemia/reperfusion
LAD: left anterior descending
lncRNA: long noncoding RNAs
MALAT1: metastasis-associated lung adenocarcinoma transcript 1
MI: myocardial infarction
NSCLC: non-small-cell lung cancer
PARP: radio immunoprecipitation assay
PI: propidium iodide
PVDF: polyvinylidene difluoride
WT: wild-type
